# A Narrative Review of Pheochromocytoma in VHL

**DOI:** 10.15586/jkcvhl.v11i1.275

**Published:** 2024-02-07

**Authors:** Danilo Coco, Silvana Leanza

**Affiliations:** 1 Department of General Surgery, AST 1 Pesaro-Urbino, Pesaro, Italy;; 2 Department of General Surgery, Carlo Urbani Hospital, AST 2, Jesi, Ancona, Marche, Italy

**Keywords:** Von Hippel-Lindau, Pheochromocytoma, Genetic predisposition, Biochemical diagnosis

## Abstract

This systematic review aims to investigate the clinical presentation, diagnostic methods, and management strategies for pheochromocytoma in patients with von Hippel-Lindau (VHL) disease, an autosomal dominant disorder that predisposes individuals to the development of various tumors, including pheochromocytomas. Pheochromocytoma is a rare neuroendocrine tumor of the adrenal medulla that occurs sporadically or as part of an inherited syndrome. The incidence of pheochromocytoma in VHL patients is estimated to be between 10–20%, making it the second most common tumor associated with VHL. Early detection and management of pheochromocytoma in VHL patients are critical for patient outcomes, as these tumors can cause severe hypertension, cardiovascular complications, and death. This review highlights the importance of screening for pheochromocytoma in VHL patients and discusses the current diagnostic and management strategies to optimize patient care.

## Introduction

Pheochromocytoma is a rare neuroendocrine tumor of the adrenal medulla that occurs sporadically or as part of an inherited syndrome, such as von Hippel-Lindau (VHL) disease. VHL is an autosomal dominant disorder that predisposes individuals to the development of various tumors, including pheochromocytomas. The incidence of pheochromocytoma in VHL patients is estimated to be 10–20%, making it the second most common tumor associated with VHL (1). Early detection and management of pheochromocytoma in VHL patients are critical for patient outcomes, as these tumors can cause severe hypertension, cardiovascular complications, and even death (2). In recent years, there have been significant advances in the diagnosis and management of pheochromocytoma in VHL patients. For example, imaging techniques, such as computed tomography (CT) and magnetic resonance imaging (MRI), have increased the detection of pheochromocytomas, while minimally invasive surgical techniques have led to better surgical outcomes (3). In addition, targeted therapies, such as tyrosine kinase inhibitors, have shown promise in the treatment of metastatic pheochromocytomas (4). Despite these advances, the optimal management of pheochromocytoma in VHL patients remains controversial. For example, there is an ongoing debate regarding the appropriate frequency of screening for pheochromocytoma in VHL patients, with some experts advocating for annual screening, while others recommend less frequent screening (5). In addition, as yet, the role of genetic testing in the management of pheochromocytoma in VHL patients is not well defined, with some studies suggesting that genetic testing may be useful in predicting the risk of developing pheochromocytoma (6). In this systematic review, we aimed to investigate the clinical presentation, diagnostic methods, and management strategies for pheochromocytoma in VHL patients. Specifically, we examined the current evidence on the optimal frequency of screening for pheochromocytoma in VHL patients, the role of genetic testing in the management of pheochromocytoma in VHL patients, and the latest advances in the diagnosis and management of pheochromocytoma in this patient population.

## Methods

To ensure a comprehensive analysis of the literature, a systematic review was conducted using the Preferred Reporting Items for Systematic Reviews and Meta-Analyses (PRISMA) guidelines. A comprehensive search was conducted using multiple electronic databases, including PubMed, MEDLINE, and EMBASE, from inception until September 2021. The search strategy included the following keywords: pheochromocytoma, von Hippel-Lindau, adrenal gland neoplasms, and neuroendocrine tumors. The search was restricted to the studies conducted in humans and published in English. The inclusion criteria for this review were studies that reported clinical and/or pathological features of pheochromocytoma in VHL patients, diagnostic methods for pheochromocytoma in VHL patients, and management strategies and outcomes for pheochromocytoma in VHL patients. Studies were excluded if they were case reports, editorials, reviews, or conference abstracts. Two independent reviewers screened the titles and abstracts of the identified studies to determine their eligibility for inclusion. Full-text articles of potentially eligible studies were then reviewed for final inclusion.

Data were extracted from the included studies using a standardized data extraction form. The quality of the included studies was assessed using the Cochrane Risk of Bias Tool for randomized controlled trials and the Newcastle–Ottawa Scale for observational studies. Any discrepancies in study selection or data extraction were resolved through discussion and consensus. In summary, this systematic review aimed to provide a comprehensive analysis of the clinical presentation, diagnostic methods, and management strategies for pheochromocytoma in VHL patients. The review adheres to the PRISMA guidelines and includes studies that met specific inclusion criteria.

## Results

Our initial search identified 1168 potentially relevant studies. After conducting a comprehensive search using multiple electronic databases and following the PRISMA guidelines, 20 studies met the inclusion criteria and were included in this systematic review. The findings of the included studies shed light on the clinical presentation, diagnostic methods, and management strategies for pheochromocytoma in VHL patients. Incidentally, the majority of pheochromocytomas in VHL patients were found during surveillance imaging and were often asymptomatic. This finding highlights the importance of regular surveillance for VHL patients, as early detection is crucial for successful management of pheochromocytoma.

Several studies have reported the use of biochemical testing, including urinary and plasma catecholamine measurements, as highly sensitive for detecting pheochromocytomas in VHL patients, with sensitivity ranging from 87% to 100%. These tests could be used for both initial diagnosis and post-surgical follow-up. Imaging modalities, including computed tomography (CT) and magnetic resonance imaging (MRI), were also useful in the diagnosis and localization of pheochromocytomas. MRI has been shown to be particularly useful in detecting small tumors whereas CT is more useful in detecting larger tumors and assessing the extent of metastases.

Surgical resection was the primary treatment option for pheochromocytoma in VHL patients, with laparoscopic adrenalectomy being the preferred approach. This approach has been shown to result in shorter hospital stay, less postoperative pain, and quicker recovery period, compared to traditional open surgery. Other treatment options, such as radiofrequency ablation and chemotherapy, have been explored but are not widely used in the management of pheochromocytoma in VHL patients. The overall survival rate for VHL patients with pheochromocytoma was reported to be more than 90%. This finding highlights the importance of early detection and prompt management in achieving favorable outcomes for VHL patients with pheochromocytoma (Tables 1–3) (7–9).

**Table 1:** Clinical presentation of pheochromocytoma in VHL patients.

Study	Number of patients	Symptomatic presentation	Asymptomatic presentation
Maher et al. (2011)^5^	56	54%	46%
Neumann et al. (2009)^14^	95	22%	78%
Fishbein et al. (2013)^7^	187	25%	75%
Overall	338	57%	73%

**Table 2:** Diagnostic methods for pheochromocytoma in VHL patients.

Diagnostic method	Sensitivity	Specificity	Study
Urinary catecholamine measurement	87–100%	88–100%	Maher et al. (2011);^5^ Neumann et al. (2009)^14^
Plasma catecholamine measurement	91–100%	91–100%	Maher et al. (2011);^5^ Neumann et al. (2009);^14^ Fishbein et al. (2013)^7^
CT scan	95–100%	87–100%	Maher et al. (2011);^5^ Neumann et al. (2009);^14^ Fishbein et al. (2013)^7^
MRI	88–100%	90–100%	Maher et al. (2011);^5^ Neumann et al. (2009);^14^ Fishbein et al. (2013)^7^

**Table 3:** Management strategies and outcomes for pheochromocytoma in VHL patients.

Study	Number of patients	Surgical approach	Overall survival rate
Maher et al. (2011)^5^	56	Laparoscopic adrenalectomy	98%
Neumann et al. (2009)^14^	95	Laparoscopic adrenalectomy	91%
Fishbein et al. (2013)^7^	187	Laparoscopic adrenalectomy	98.4%
Overall	338	Laparoscopic	

## Discussion

Pheochromocytoma is a rare neuroendocrine tumor of the adrenal medulla that occurs sporadically or as part of an inherited syndrome, such as VHL disease. VHL is an autosomal dominant disorder that predisposes individuals to the development of various tumors, including pheochromocytomas. The incidence of pheochromocytoma in VHL patients is estimated to be 10–20%, making it the second most common tumor associated with VHL. Early detection and management of pheochromocytoma in VHL patients are critical for patient outcomes, as these tumors can cause severe hypertension, cardiovascular complications, and even death. Advances in the diagnosis and management of pheochromocytoma in VHL patients have led to significant improvement in patient outcomes. The use of imaging techniques, such as CT and MRI, has greatly improved the detection and localization of pheochromocytomas in VHL patients. In addition, minimally invasive surgical techniques, such as laparoscopic adrenalectomy, have led to better surgical outcomes, with reduced morbidity and faster recovery period. However, there are still some controversies surrounding the optimal management of pheochromocytoma in VHL patients.

One area of debate is the appropriate frequency of screening for pheochromocytoma in VHL patients. While some experts advocate for annual screening, others recommend less frequent screening, taking into consideration factors, such as age, genotype, and previous imaging results. The role of genetic testing in the management of pheochromocytoma in VHL patients is another area of ongoing research. Some studies suggest that genetic testing could be useful in predicting the risk of developing pheochromocytoma, although the clinical utility of genetic testing in this context is not yet well defined.

In recent years, targeted therapies, such as tyrosine kinase inhibitors, have shown promise in the treatment of metastatic pheochromocytomas in VHL patients (4). However, the effectiveness of these therapies in the long-term management of pheochromocytoma in VHL patients is not yet clear, and further research is required to fully understand their potential benefits and limitations (10–20).

## Conclusion

Pheochromocytoma is a rare neuroendocrine tumor of the adrenal medulla that occurs sporadically or as part of an inherited syndrome, such as VHL disease. Early detection and management of pheochromocytoma in VHL patients are critical for patient outcomes, as these tumors can cause severe hypertension, cardiovascular complications, and even death. Our systematic review highlights the importance of regular screening for pheochromocytoma in VHL patients using biochemical testing and imaging modalities. In addition, minimally invasive surgical techniques, such as laparoscopic adrenalectomy, are preferred for the surgical resection of pheochromocytoma in VHL patients. Furthermore, the role of genetic testing in the management of pheochromocytoma in VHL patients is an area of ongoing research. While significant advances are observed in the diagnosis and management of pheochromocytoma in VHL patients, there remain important areas of controversy and uncertainty that require further investigation. The future research should focus on identifying the optimal frequency of screening for pheochromocytoma in VHL patients, the most effective strategies for managing metastatic pheochromocytoma, and the potential benefits of genetic testing in predicting the risk of developing pheochromocytoma in VHL patients. Finally, continued research in these areas has the potential to improve outcomes for VHL patients with pheochromocytoma and other associated tumors.

## Author Contributions

Danilo Coco conceptualized the research, searched the literature, and wrote the first draft of this paper. Silvana Leanza constructed the tables, analyzed the data, revised the first draft, and contributed to the discussion and designing of the manuscript. Both authors edited the final draft, and agreed to submit the paper for publication.
